# How to utilize routine antimicrobial resistance surveillance data for local and national actions in an LMIC

**DOI:** 10.1093/jacamr/dlaf225

**Published:** 2025-12-02

**Authors:** Panida Chamawan, Direk Limmathurotsakul

**Affiliations:** The Office of Permanent Secretary, Ministry of Public Health, Nonthaburi, Thailand; Mahidol Oxford Tropical Medicine Research Unit, Faculty of Tropical Medicine, Mahidol University, Bangkok, Thailand; Nuffield Department of Medicine, Centre for Tropical Medicine and Global Health, University of Oxford, Oxford, UK; Department of Tropical Hygiene, Faculty of Tropical Medicine, Mahidol University, Bangkok, Thailand

## Abstract

We describe here how we guide and work with over 100 secondary- and tertiary-care hospitals in Thailand to support the effective use of their antimicrobial resistance (AMR) surveillance data for local actions, and with policy makers for national actions. At the facility level, the guidance includes: (i) validating data, (ii) comparing data with previous reports, (iii) comparing data with other hospitals with similar levels of care and bed count, (iv) comparing cluster signals with infection prevention control records, and (v) identifying wards with hyperendemic hospital-origin AMR infections. At the national level, the guidance includes monitoring national estimates, systematically benchmarking hospital-level estimates, and developing national guidelines for empirical antimicrobial therapy. We encourage hospitals and policy makers in other low- and middle-income countries to explore, adopt and adapt this guidance, ensuring their AMR surveillance data are effectively used for their local and national actions based on their context and constraints.

## Introduction

The WHO Global Antimicrobial Resistance Surveillance System (GLASS) has set out to standardize the collection, analysis, interpretation and sharing of antimicrobial resistance (AMR) surveillance data across countries.^1^ The growing volume of global data provides an opportunity for global actions.^1^ Nonetheless, there remains limited guidance for healthcare facilities and policy makers in low- and middle-income countries (LMICs) on how to use the collected data for local and national actions. This limitation is probably due to several factors, including variability in data availability and quality,^2,3^ risk of misinterpreting the poor-quality data, potential biases caused by the underuse of bacterial culture in routine practice,^4^ and challenges in data interpretation.^1^

**Figure 1.**
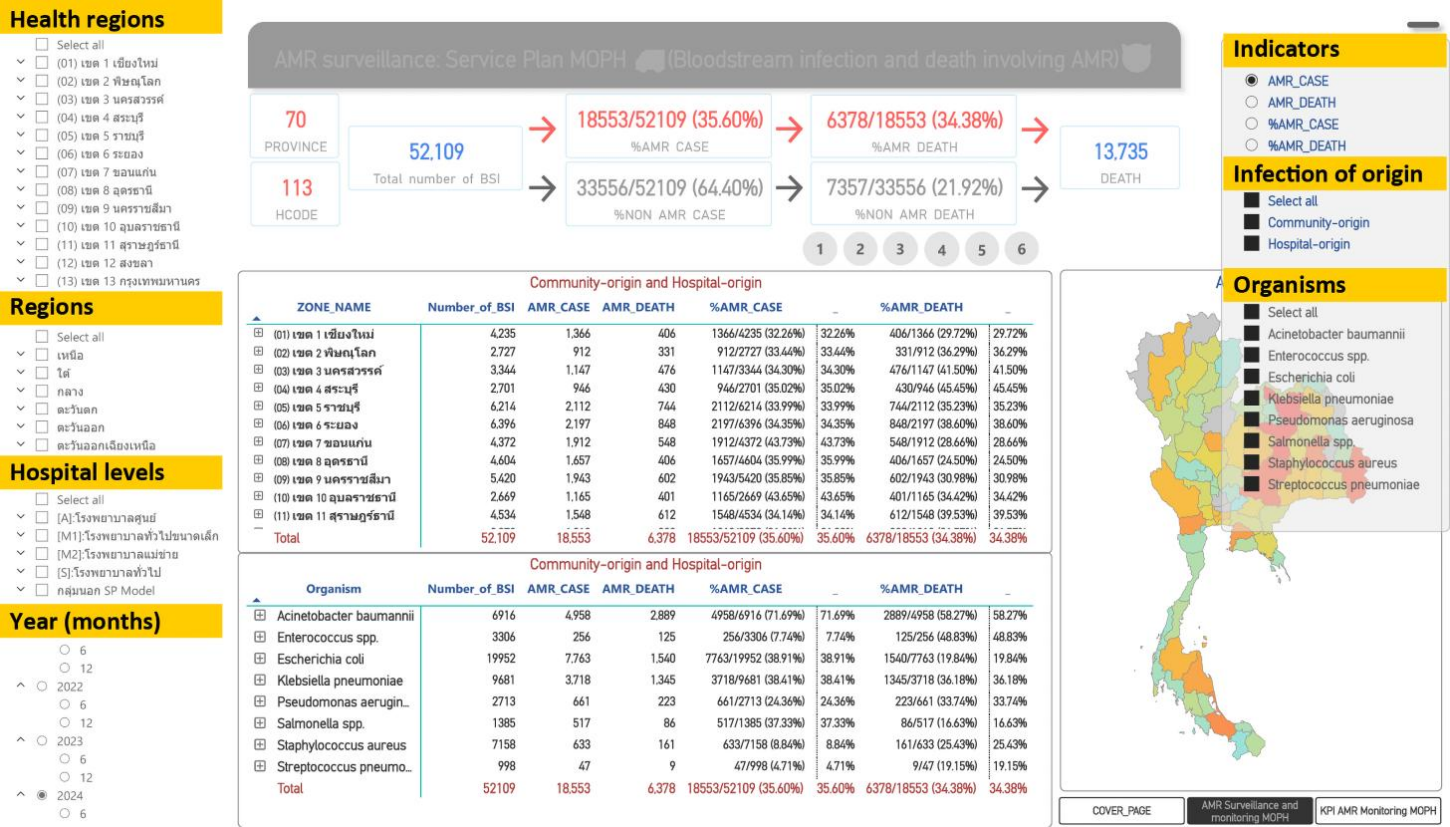
New interactive Thailand AMR dashboard [Thai words translated into English are highlighted in yellow]. The picture was captured from the website^10^ on 15 June 2025 representing data of the year 2024 from 113 public hospitals in Thailand.

Here, we describe how the Health Administration Division of the Ministry of Public Health (MoPH), Thailand, has been guiding and working with 127 public secondary- and tertiary-care hospitals (SCHs and TCHs) to support the use of their AMR surveillance data at both facility and national levels.^5,6^ In 2022–2023, we started by training 26 and expanded to all 127 SCHs and TCHs under the MoPH to use the AutoMated tool for Antimicrobial resistance Surveillance System (AMASS) so that all hospitals can independently generate their own AMR surveillance reports (in pdf and Excel formats), validate their summary data, and use their data for local actions.^5,6^ AMASS is an offline, open-access application that allows a facility to generate both isolate-based and sample-based reports stratified by infection origin from their raw data files,^7^ using the analytical algorithms adopted from WHO GLASS. In 2022, the Department of Medical Science, MoPH, collected raw data files from 16 facilities and generated national estimates for WHO GLASS.^8,9^ The data submitted to WHO GLASS were not used in the development of this guidance. Since 2023, all 127 public SCHs and TCHs under the MoPH have been supported to use AMASS and submit the facility-level summary data (in Excel format) to the MoPH every 6 months. Once submitted, these data are made available in real time on the MoPH dashboard (Figure 1).^6,10^

## Guidance

### At the facility level

After generating an AMR surveillance report (Table 1), each facility should:

**Table 1.** How to utilize AMR surveillance data at the facility level

Key guidance	Sampled key parameters^a^	Sampled key questions^a^
(a) Validate data	Total number of patients with CO BSI, HO BSI and BSI of unknown origin caused by each AMR priority pathogen Total number of patients tested for CO BSI and HO BSI AMR proportion and frequency of CO BSI and HO BSI caused by each AMR priority pathogen In-hospital mortality (%) and total number of in-hospital deaths (*n*) among inpatients with CO BSI and HO BSI caused by each AMR priority pathogen^b^	Over the past year at my hospital, how many patients had CO BSI, HO BSI and BSI of unknown origin caused by each AMR priority pathogen? How many blood culture specimens were processed by the microbiology laboratory? After de-duplication and proper analysis, how many patients were tested for CO BSI and HO BSI? What was the AMR proportion for each AMR priority pathogen? How many patients had CO and HO BSI caused by each AMR priority pathogen among 100 000 patients tested for CO BSI and HO BSI, respectively (i.e. AMR frequency per 100 000 tested patients)? How many inpatients were recorded to have died following CO BSI and HO BSI caused by each AMR priority pathogen?^b^ After generating the complete facility-level summary data, do these numbers and estimates accurately reflect the real situation observed by clinicians, microbiologists, and the AMS and IPC teams?
(b) Compare data with previous reports	(similar to above)	Over the past year at my hospital, have the total number of patients having HO BSI caused by CRAB increased or decreased compared with the previous years? Have these numbers and estimates for each AMR priority pathogen increased or decreased compared with previous years? If a considerable increase or decrease is observed, does it truly represent a change in the AMR burden at my hospital? Was it caused by changes in AMS, IPC or other factors unrelated to the actual AMR burden (e.g. changes in diagnostic stewardship practices, modifications in laboratory procedures, updates to breakpoints, and shortages of AST discs)?
(c) Compare data with other hospitals with similar levels of care and bed count	(similar to above)	Over the past year at my hospital, was the total number of patients having HO BSI caused by CRAB higher or lower compared with other hospitals with similar levels of care and bed count? Were these numbers and estimates higher or lower than other hospitals? If there is a considerable difference in indicators for HO AMR infections, could it be caused by variations in AMS, IPC, patient case mix, or other factors unrelated to the actual AMR burden? If there is a considerable difference in indicators for CO AMR infections, could it be caused by differences in WASH conditions, adherence to vaccination programmes, population characteristics, disease endemicity or other factors unrelated to the actual AMR burden?
(d) Compare cluster signals with IPC team records	Cluster signals of patients with HO BSI caused by each AMR priority pathogen^c^	Over the past year at my hospital, were cluster signals retrospectively detected by the AODS similar or different from the IPC team records? If there is a considerable difference, should the IPC team conduct a situation analysis to define whether signals are false-positive or false-negative? Could this situation analysis inform whether the current cluster detection methods routinely used by the IPC team need to be modified?
(e) Identify wards with hyperendemic HO AMR infections	Total number of new patients with HO infections caused by each AMR priority pathogen by each ward	Over the past year at my hospital, which three wards had the highest number of new patients with HO infections caused by each AMR priority pathogen? Were there probably hyperendemic HO infections in these wards? What are the barriers to IPC and AMS practices in these wards, if any? Should AMS and IPC practices be strengthened in these wards? If so, what specific interventions should be implemented?

AMR, antimicrobial resistance; AMS, Antimicrobial Stewardship; AODS, Automated Outbreak Detection System; AST, antimicrobial susceptibility testing; BSI, bloodstream infection; CO, community-origin; CRAB, carbapenem-resistant *Acinetobacter baumannii*; HO, hospital-origin; IPC, Infection Prevention and Control; WASH, water, sanitation and hygiene.

^a^Many parameters and questions may not be feasible at the facility level if data are not recorded or available. For example, the total number of patients tested for CO BSI and HO BSI could be estimated if data of all blood culture specimens are electronically recorded and if hospital admission date data are electronically recorded in data files.

^b^In-hospital mortality represents all-cause mortality. The mortality of a proportion of patients with AMR infections might be due to other causes of death such as exacerbation of underlying disease. In addition, a proportion of patients might prefer to die at home, and this proportion may be different between regions in a country. Nonetheless, the crude total number of in-hospital deaths is still important for local healthcare officers and policy makers to understand the situation within their healthcare facilities.

^c^Cluster signal analysis can be performed by SaTScan (integrated into WHONET)^11^ and other automated outbreak detection systems.^12^ SaTScan is also integrated into AMASS (AutoMated tool for Antimicrobial resistance Surveillance System), an open-access, off-line and easy-to-use automated tool that has been implemented in >100 secondary- and tertiary-care hospitals in Thailand.^5,6^

**1. Validate data.** Summary AMR data (whether generated by AMASS, WHONET or any other software) should be validated by comparison with manually calculated numbers derived from the complete line listing of several organisms.^13^ Baseline characteristic data should also be cross-checked against hospital workload indicators (e.g. total number of hospital admissions and total number of blood culture specimens). Key stakeholders at the facility, including members of the Antimicrobial Stewardship (AMS) and Infection Prevention and Control (IPC) teams, should assess whether the summary data appear accurate (Table 1). If discrepancies are suspected, they should be investigated by reviewing the raw data and regenerating summary data (if needed).

**2. Compare data with previous reports.** For each priority AMR pathogen [e.g. carbapenem-resistant *Acinetobacter baumannii* (CRAB)], facilities should monitor changes across all numbers and estimates over time. Facilities must consider whether the observed changes could be caused by other factors unrelated to the actual AMR burden.

Attention should be paid to changes that may have clinical significance. For example, in a 1000-bed hospital, if the proportion of CRAB among patients with hospital-origin bloodstream infection (BSI) caused by *A. baumannii* remains stable at 90% (e.g. 90/100 patients in 2022 and 180/200 patients in 2023), and both the number of hospital admissions per year (∼60 000) and the number of patients tested for hospital-origin BSI per year (∼3000) remain largely stable, then an increase in the frequency of hospital-origin CRAB BSI from 3000 to 6000 per 100 000 tested patients is a critical change. This increase reflects a true rise in the number of patients with hospital-origin CRAB BSI from 90 in 2022 to 180 in 2023 and warrants immediate attention.

To facilitate communication, we also simplify the concept of AMR frequency. For example, a frequency of 3000 hospital-origin CRAB BSI per 100 000 patients tested for hospital-origin BSI can be explained as, ‘In your hospital, out of every 100 patients who had blood samples collected and cultured for suspected hospital-origin BSI, about 3 were blood culture positive for CRAB (i.e. about 3%).’

**3. Compare data with other hospitals with similar levels of care and bed count.** For each priority AMR pathogen, facilities should compare all numbers and estimates with those from other hospitals.

Attention should also be paid to differences that may have clinical significance. For example, many small 200-bed SCHs may traditionally not report the proportion of CRAB from blood specimens due to having fewer than 30 cases of *A. baumannii* BSI.^13^ However, we advocate for the systematic reporting of all numbers and estimates together with their 95% CIs. We note potential uses of good quality data even from small sample sizes. Consider a scenario where a 200-bed SCH reports a 50% proportion of CRAB from blood specimens (10/20 patients), whereas other SCHs with similar size in the same region report proportions of 0%, 50% or 100% based on very low case numbers (e.g. 0/2, 1/2 or 2/2 patients, respectively). This pattern indicates a potential outbreak or hyperendemicity of CRAB BSI at the facility with 10 patients with CRAB BSI, and warrants immediate attention.

**Figure 2.**
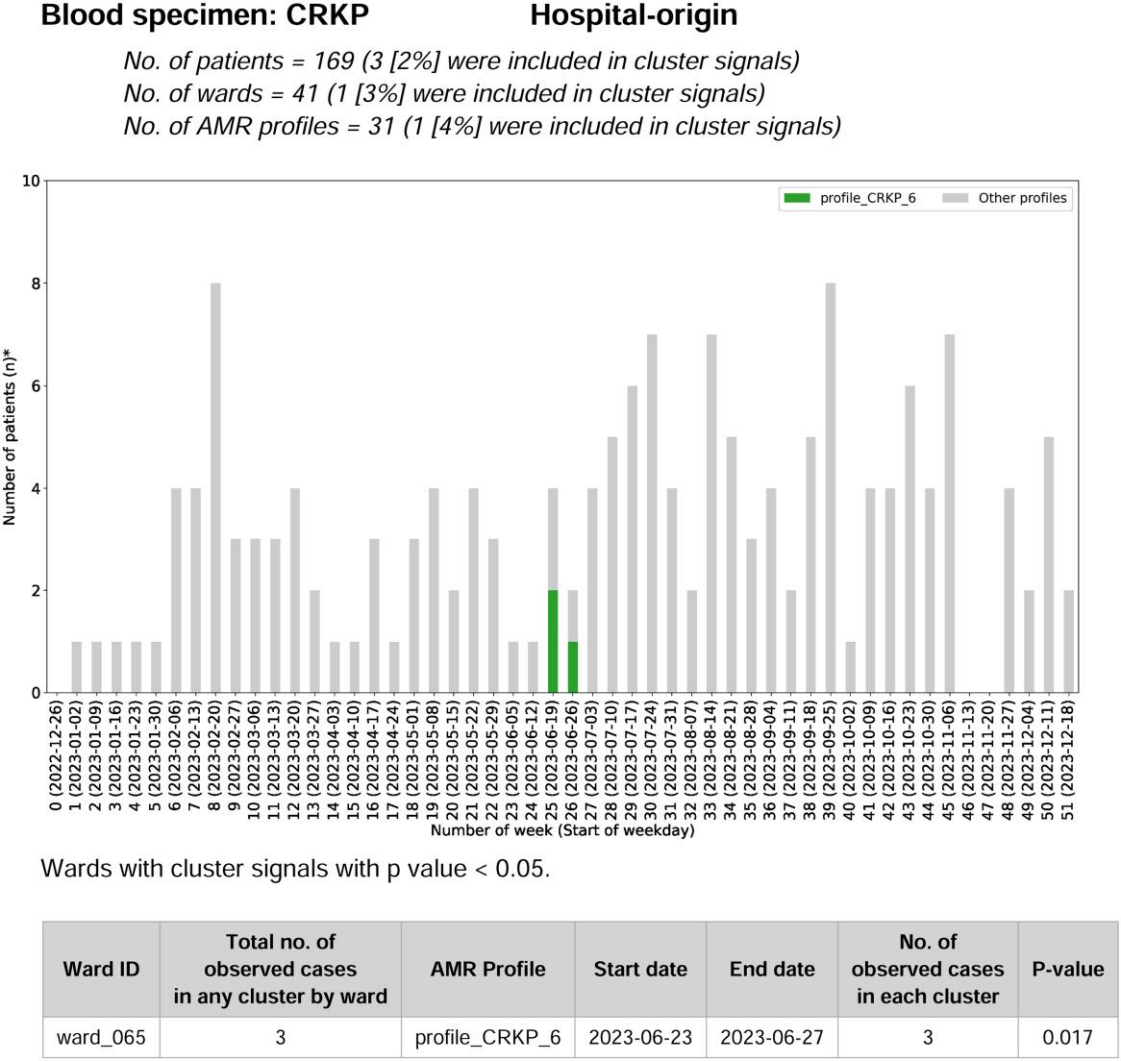
Example of cluster signals automatically generated and reported by AMASS. In this example, a cluster of hospital-origin carbapenem-resistant *Klebsiella pneumoniae* (CRKP) bloodstream infection was detected in ward code 065 (ward_065) during the period from 23 to 27 June 2023. The CRKP in the cluster had drug resistance profile 6 (profile_CRKP_6). A total of three patients were involved in this cluster.

**4. Compare data cluster signal analysis with IPC team records.** AMR surveillance reports may include cluster signals *retrospectively* generated from automated outbreak detection systems (AODS) using various analytical algorithms.^12^ SaTScan is increasingly used,^14,15^ and readily integrated in WHONET^11,16^ and AMASS (Figure 2).

Clusters identified by both AODS and IPC teams can serve as mutual confirmation. Discrepancies may prompt IPC teams to conduct further investigations to determine whether the signals represent true or false positives, and whether the existing outbreak detection methods (e.g. simple thresholds^12^) need modification. Facilities with capabilities can also use AODS to *prospectively* generate real-time cluster signal alerts.^15^

**5. Identify wards with hyperendemic hospital-origin AMR infections.** This can help AMS and IPC teams prioritize wards where interventions are mostly needed.

Hyperendemicity, in general, refers to the persistent occurrence of a disease within a population at a consistently high incidence rate.^17^ For practical purposes, we suggest that wards with the highest number of new patients with hospital-origin infections caused by each priority AMR pathogen can be initially suspected of hyperendemicity. Further investigations can be conducted using the frequency of hospital-origin infections per 100 000 bed-days (when possible) and clinical data. We note that hyperendemicity may not be detected as clusters because a cluster represents a short-term spike in incidence above a baseline, whereas hyperendemicity represents a consistently high incidence rate over time.

We also note that further investigations using clinical data may suggest that some patients possibly acquired the infections at transferring wards or hospitals. However, such findings should not undermine the need for improvement. Instead, all involved wards and hospitals should collaboratively consider additional measures (e.g. screening for AMR colonization or infection before admission to high-risk wards, and improving transfer protocols to include information of AMR status and required IPC procedures).

### At the national level

After compiling facility-level summary AMR surveillance data (Table 2), national authorities should:

**Table 2.** How to utilize AMR surveillance data at the national level

Key guidance	Sampled key parameters^a^	Sampled key questions^a^
(a) Validate data and generate national estimates	Total number of patients with CO BSI and HO BSI by each AMR priority pathogen Total number of patients tested for CO BSI and HO BSI Total number of in-hospital deaths (n) among inpatients with CO BSI and HO BSI caused by each AMR priority pathogen National estimates of AMR proportion and frequency of CO BSI and HO BSI caused by each AMR priority pathogen National estimates of the total number of deaths attributable to AMR infection	Over the past year in my country, how many patients were reported as having CO BSI and HO BSI caused by each AMR priority pathogen? How many patients were reported to have died following CO BSI and HO BSI caused by each AMR priority pathogen? After generating the complete set of national estimates, do these national estimates accurately reflect the real situation observed by national AMR committee and healthcare workers in my country? Using statistical or modelling methods,^18,19^ how many deaths were estimated to be attributable to AMR infection in my country (when possible)?
(b) Compare national estimates with previous reports	(similar to above)	Over the past year in my country, have these national estimates increased or decreased compared with previous years? If a considerable increase or decrease is observed, does it truly represent a change in the national AMR burden? Was it caused by changes in national policies on AMS and IPC, national interventions or other factors unrelated to the actual AMR burden (e.g. changes in national guidelines for diagnostic stewardship, and the expansion of surveillance sites)? Has the national estimate of deaths attributable to AMR infection increased or decreased compared with the previous years?
(c) Systematically benchmark between facilities with similar levels of care and bed count	(similar to above)	Is there a considerable difference in facility-level indicators for *HO AMR infection* between facilities with similar levels of care and bed count? If yes, should policy makers prioritize interventions in *facilities* with the highest burden? Is there a considerable difference in facility-level indicators for *CO AMR infection* between facilities with similar levels of care and bed count? If yes, should policy makers prioritize interventions in *geographical areas* (including communities and facilities) with the highest burden?
(d) Monitor emerging AMR pathogen	Presence of emerging or exceptional AMR phenotypes?	Over the past year in my country, have any facilities observed emerging or exceptional AMR phenotypes (e.g. carbapenem-resistant *Salmonella* spp.)? If yes, did the national authority (e.g. national laboratory reference centre) provide support for isolate collection and further investigation? If confirmed, did the national authority report and communicate the findings in accordance with international agreements?^20^
(e) Develop guidelines for empirical antimicrobial therapy	Guidelines for empirical antimicrobial therapy for each clinical syndrome	Do we have guidelines for empirical antimicrobial therapy for all clinical syndromes? If yes, are they based on the most updated national estimates and data? Should any guidelines be revised or updated?

AMR, antimicrobial resistance; BSI, bloodstream infection; CO, community-origin; HO, hospital-origin.

^a^ Many parameters and questions may not be feasible at the national level if data are not recorded, available or submitted. For example, the total number of in-hospital deaths among inpatients with CO BSI and HO BSI caused by each AMR priority pathogen may not be available if data are not recorded at the facility level.

**1. Validate data and generate national estimates.** National authorities must thoroughly review each facility-level summary data submitted, and cross-check with facilities as needed to ensure data accuracy. Additionally, national authorities should carefully determine appropriate methodologies for generating national estimates; e.g. simple data aggregation, the use of medians or other statistical methods, and whether to apply weighting based on hospital levels of care and other factors.^21^

**2. Compare national estimates with previous reports.** Changes in national estimates enable policy makers to evaluate the impact of national interventions and whether the national AMR burden is improving or deteriorating. Nonetheless, national authorities need to consider whether the observed changes could be caused by other factors unrelated to the actual AMR burden.

We support the use of individual-level and facility-level AMR surveillance data to estimate the total number of deaths attributable to AMR infections within the country by national organizations and through multiple analytical methods.^18,19^ This estimate could be used for tracking progress in reducing the total number of AMR-related deaths by 10% by 2030.^22^ We also support the comparison of national estimates with those generated by international organizations,^23^ while carefully evaluating any similarities and discrepancies in data sources, analytical methods and underlying assumptions.

**Figure 3.**
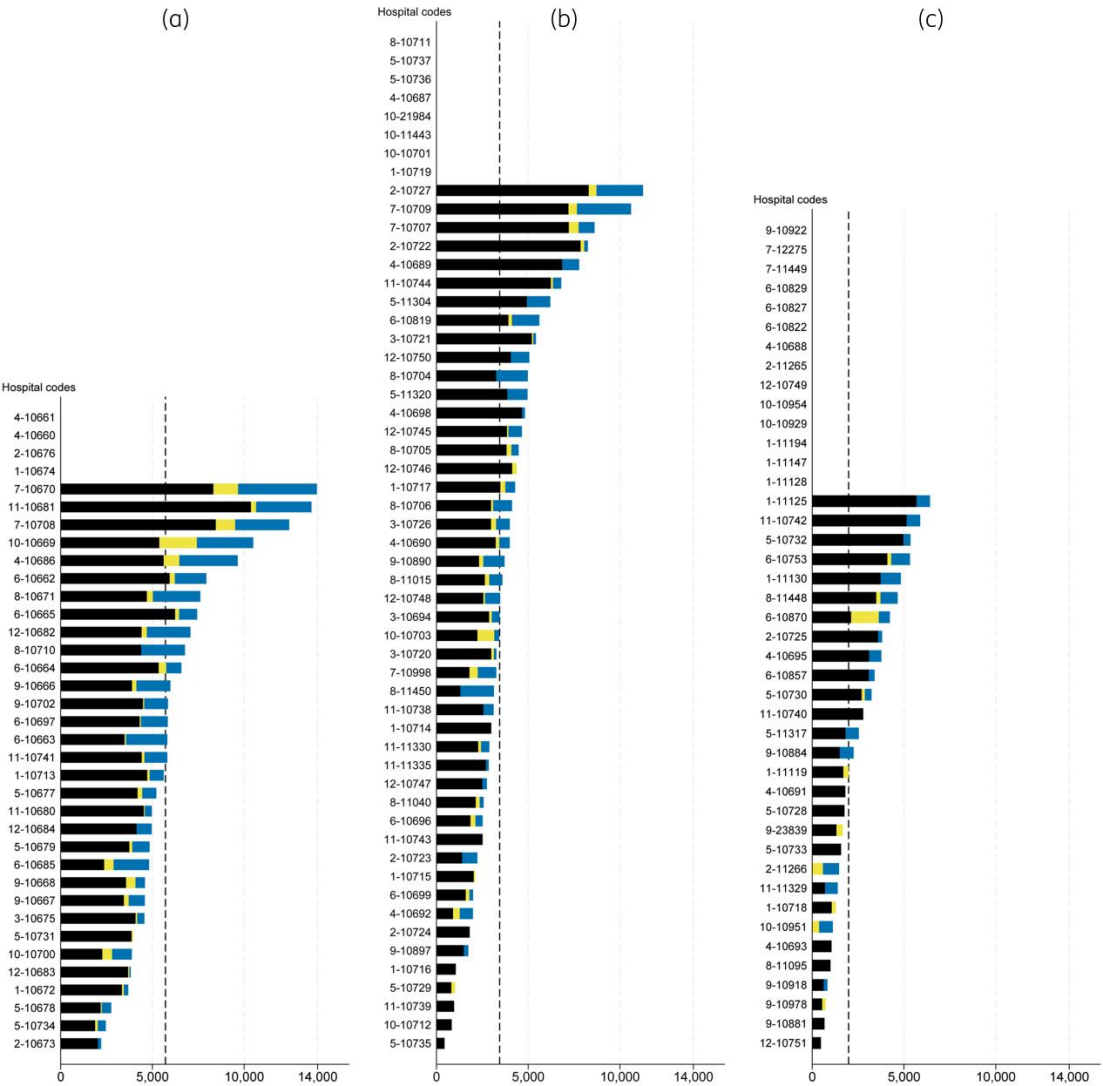
Example comparison of the frequency of hospital-origin (HO) antimicrobial-resistant (AMR) infections between facilities with similar levels of care. This figure compares the frequency of HO bloodstream infections (BSIs) caused by carbapenem-resistant *Acinetobacter baumannii* (CRAB), *Escherichia coli* (CREC) and *Klebsiella pneumoniae* (CRKP) per 100 000 patients tested for HO BSI across three hospital levels: advanced-level [Level A (a)], standard-level [Level S (b)] and mid-level [Level M1 (c)] referral hospitals. Frequencies of HO CRAB, CREC and CRKP BSI are represented in black, yellow and blue, respectively. Data are of the year 2024 as of 15 June 2025. Hospitals without submitted data are listed at the top of the figures. Dashed lines indicate the median frequency within each hospital level. In 2024, the Health Administration Division, Ministry of Public Health (MoPH) supervised 134 public referral hospitals in health regions 1 to 12, including 36 Level A hospitals (approximately 500–1200 beds), 55 Level S hospitals (approximately 300–500 beds) and 43 Level M1 hospitals (approximately 180–300 beds).^24^

**3. Systematically benchmark data between facilities with similar levels of care and bed count.** Comparing indicators of *hospital-origin AMR infection* enables policy makers to classify facilities based on their burden (Figure 3). We recommend comparing all indicators including total number of cases, total number of tested patients, and AMR frequency, not just AMR proportion. National authorities could prioritize monitoring, audits and support for AMS and IPC practices at hospitals with the highest burden.^5,6^ National authorities could also cross-check and acknowledge hospitals with the lowest burden as ‘champions’ or ‘mentors’, providing support and fostering collaborative improvement between facilities.^5,6^

Comparing indicators of *community-origin AMR infection* enables policy makers to identify geographical areas based on their burden. Geographical areas (including communities and facilities) with the highest burden could be considered for further investigations and targeted interventions, e.g. improvements in water, sanitation and hygiene (WASH), community-based AMS programmes, and vaccination initiatives.

Comparing *availability and quality of data* across facilities enables policy makers to identify issues that may require targeted interventions or support, particularly among hospitals where summary data could not be generated and submitted. For example, during the implementation of AMASS in Thailand, we observed and then evaluated diversity in Laboratory Information Management Systems (LIMS), Hospital Information Systems (HIS) and data management practices.^25^ Currently, we are developing and providing recommendations to improve information systems and data management practices at both facility and national levels.

**4. Monitor emerging AMR pathogens.** National authorities should support the monitoring of emerging or exceptional AMR phenotypes (e.g. carbapenem-resistant *Salmonella* spp.).^20^ When such phenotypes are suspected, national authorities must support the collection of bacterial isolates and relevant clinical data for genotyping and further investigation, respectively. Once confirmed by the national authorities, novel AMR genotypes with potential public health implications should be promptly reported and communicated in accordance with international agreements.^20^

**5. Develop guidelines for empirical antimicrobial therapy**. We recommend that guidelines for empirical antimicrobial therapy should be developed at the national level to ensure consistency and reliability. This is because AMR surveillance data from each individual hospital may be biased due to small sample sizes and, in some settings, due to a high proportion of patients being sampled only after empirical treatment has failed.^2–4^ The development of national antibiotic prescribing guidelines should involve a multidisciplinary committee, including infectious disease specialists, microbiologists, pharmacists and public health epidemiologists. In case that recommendations needed to be modified based on hospital levels or diseases endemic in a region, the modification should also be done at the national level.^26^ For example, *Burkholderia pseudomallei*, the pathogen causing melioidosis, is intrinsically resistant to most third-generation cephalosporins. The national guideline for melioidosis recommends including ceftazidime in empirical antimicrobial therapy for patients presenting with community-acquired sepsis who have a risk factor for melioidosis, in regions where the disease is endemic.^26^

## Discussion

Here, we present a short and practical guidance based on our context in Thailand, where data availability, data quality and diagnostic stewardship practices are relatively good compared with other LMICs,^4,27^ LIMS and HIS are widely available but often lack connectivity,^25^ AMS practices remain limited,^28^ and the burden of AMR is high.^6^ Enhancing the use of surveillance data would support the monitoring and evaluation of our AMR interventions at both facility and national levels.

The challenge of how to utilize AMR surveillance data for actions in LMICs still lacks research and evidence. Our guidance is grounded in practical experience rather than being fully evidence-based, and may not be directly applicable to other LMIC contexts. Research funders are encouraged to prioritize this gap when outlining AMR funding calls.

Overall, we encourage hospitals and policy makers in other LMICs to explore, adopt and adapt this guidance, ensuring that their AMR surveillance data are effectively utilized for actions based on their context and constraints. Some settings may find that their AMR surveillance data are of limited quality,^3^ and initial efforts could include strengthening diagnostic stewardship,^27^ laboratory capacity and quality, and surveillance programmes.^29^
